# Highly inclined light sheet allows volumetric super-resolution imaging of efflux pumps distribution in bacterial biofilms

**DOI:** 10.1038/s41598-024-63729-x

**Published:** 2024-06-05

**Authors:** T. Vignolini, M. Capitanio, C. Caldini, L. Gardini, F. S. Pavone

**Affiliations:** 1 European Laboratory for Non- Linear Spectroscopy, LENS, Via N. Carrara 1, 50019 Sesto Fiorentino, Italy; 2 Department of Physics and Astronomy, University of Florence, Via G. Sansone 1, 50019 Sesto Fiorentino, Italy; 3 National Institute of Optics, National Research Council, Via N. Carrara 1, 50019 Sesto Fiorentino, Italy; 4 Present Address: Parasite RNA Biology Group, Institut Pasteur, Université Paris Cité, 75015 Paris, France

**Keywords:** Single-molecule biophysics, Biofilms

## Abstract

Bacterial biofilms are highly complex communities in which isogenic bacteria display different gene expression patterns and organize in a three-dimensional mesh gaining enhanced resistance to biocides. The molecular mechanisms behind such increased resistance remain mostly unknown, also because of the technical difficulties in biofilm investigation at the sub-cellular and molecular level. In this work we focus on the AcrAB-TolC protein complex, a multidrug efflux pump found in Enterobacteriaceae, whose overexpression is associated with most multiple drug resistance (MDR) phenotypes occurring in Gram-negative bacteria. We propose an optical method to quantify the expression level of the AcrAB-TolC pump within the biofilm volume at the sub-cellular level, with single-molecule sensitivity. Through a combination of super-resolution PALM with single objective light sheet and precision genome editing, we can directly quantify the spatial distribution of endogenous AcrAB-TolC pumps expressed in both planktonic bacteria and, importantly, within the bacterial biofilm volume. We observe a gradient of pump density within the biofilm volume and over the course of biofilm maturation. Notably, we propose an optical method that could be broadly employed to achieve volumetric super-resolution imaging of thick samples.

## Introduction

Antimicrobial resistance in pathogenic bacteria is a growing, worldwide health concern. As much as 50,000 people die prematurely every year due to antimicrobial-resistant infections in Europe and in the US alone^1^. One of the most successful resistance strategies adopted by bacteria against antimicrobial treatments is the expression of efflux pumps (EPs), i.e., a class of transporter proteins capable of extruding a wide range of molecules from bacteria such as harmful metabolic products or xenobiotics^2^. Gram-negative bacteria multiple drug resistance (MDR), is mostly associated with the overexpression of one or multiple types of EPs, mainly belonging to the Resistance-Nodulation-cell Division (RND) superfamily^3,4^.

A model system for RND efflux pumps is the acridine resistance complex of *Escherichia coli*^5,6^, named AcrAB-TolC. This is an assembly of proteins spanning both membranes and driven by proton motive force, composed by three different proteins: AcrB, an antiporter with broad substrate specificity which spans the cytoplasmic membrane; TolC, an outer membrane channel; and AcrA, a periplasmic adapter^7,8^. Although AcrAB-TolC is only found in Enterobacteriaceae such as *E. coli*, *Salmonella* species and *Klebsiella pneumoniae*^9^, homologous RND efflux pumps are found in many other species of Gram-negative bacteria^5,9–16^.

Another strategy adopted by bacteria to achieve a high degree of resistance to antimicrobial treatments is to become enmeshed in biofilms. Biofilm-associated bacteria have been found to be up to 2500 times more resistant to antibiotics than their freely swimming counterparts^17^, and the mechanisms underlying this extreme resiliency are still unclear. On one side, intercellular space in biofilms is filled with a viscous matrix composed of exopolysaccharides (EPS), proteins, and extracellular nucleic acids (eDNA and eRNA), which plays a role in limiting the diffusion of biocides^18,19^. On the other side, biofilm resistance to antibiotics has been linked to an increased expression of EPs^20,21^, as the use of EP inhibitors on biofilms has been shown to significantly decrease their resistance against drugs^21,22^. Biofilms are highly complex structures where isogenic bacteria display different gene expression patterns due to intercellular interactions (quorum sensing mechanisms), extracellular concentration gradients, and stochastic fluctuations^23^. These features make investigating the role of EPs and their expression dynamics in biofilms technically challenging. In fact, currently available data on EPs expression in bacterial populations is mostly based on mRNA extraction from bulk samples or on transcriptional fusions to reporter genes, which provide averaged values from pools of billions of individual cells and no spatial information^21,24–28^. On the contrary, single-molecule techniques would allow direct measurement of the spatial and temporal distribution of EPs within the biofilm volume and would give new insights in their possible role in the increased antimicrobial resistance displayed by these bacterial communities.

Here we demonstrate that through a combination of PhotoActivated Localization Microscopy (PALM) with custom illumination geometry^29^ we can achieve single-molecule sensitivity deep in the biofilm volume, and thanks to precise genome editing we can use this approach to directly visualize and quantify the levels of expression of endogenous EPs during biofilm maturation.

## Results

**Figure 1:**
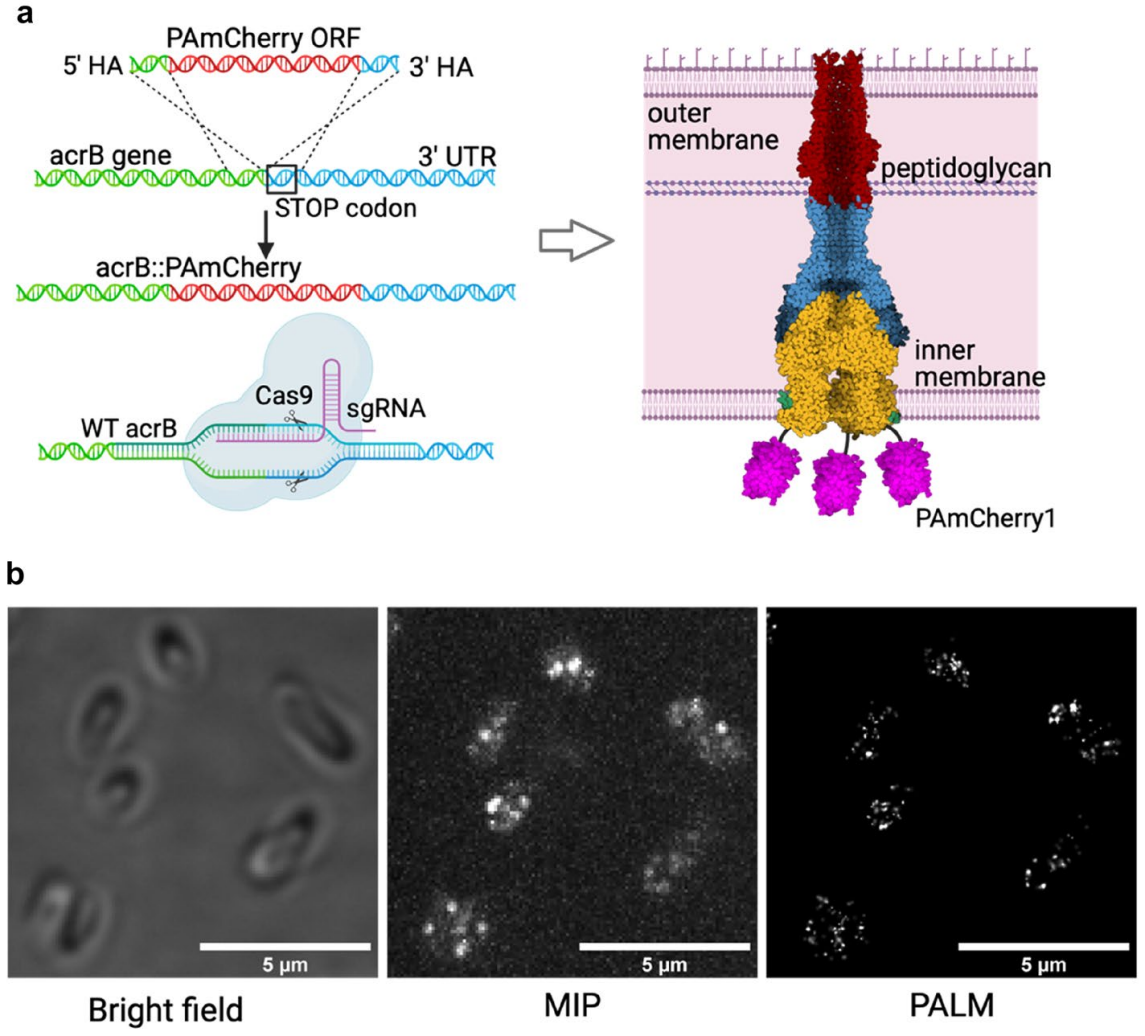
(**a**) Genome editing approach used to produce E.*coli* strain with endogenous expression of the efflux pump AcrAB-TolC labelled with PAmCherry1. Left: schematic of the genome editing process used in the production of *E. coli* TV001 strain. A double-stranded DNA template encoding for PAmCherry1 was recombined into the *E. coli* BW25113 chromosome and positive recombinants were selected via a CRISPR/Cas9 system targeting the WT chromosomal locus. Right: schematic of the labelling configuration. Each AcrB monomer in the pump is fused with a C-terminal PAmCherry1, which leads to every complete pump being labelled with three copies of the tag. Created with BioRender.com (**b**) Imaging of the genetically modified planktonic E.*coli* strain. Left panel: example of bright field image of planktonic of *E. coli* TV001. Middle panel: maximum intensity projections (MIP) generated from 1000 frames of a PALM acquisition under simultaneous illumination with green and violet light. Right panel: super-resolved image reconstructed with ThunderSTORM software^44^.

In order to measure the spatial distribution of AcrAB-TolC EP within bacterial biofilms, we designed a labelling strategy for endogenous AcrB instances, i.e. the intracellular component of the AcrAB-TolC EP (Fig. 1a). The gene encoding for AcrB in the chromosome of *E. coli* strain BW25113 was edited with the insertion of an in-frame sequence encoding for the fluorescent photoactivatable protein PAmCherry1^30^, which replaced the “stop” codon of the original AcrB open reading frame (ORF). The N-terminal of the fluorescent protein was designed to be linked with a short flexible peptide (Gly–Gly–Gly) with the C-terminal of AcrB, as previously described for a GFP fusion by Yamamoto et al.^31^ (Fig. 1a). To ensure minimal influence on the expression of the labelled protein the insert sequence was custom-synthesized with an optimized codon bias for *E. coli* BW25113 and genome editing was performed with a slight variation of the no-SCAR protocol by Reisch and Prather^32^, which allows the arbitrary editing of genomic DNA without the addition of any extra bases that could affect transcription. The no-SCAR method is a λ Red recombination-mediated genetic engineering approach (a.k.a. “recombineering”^33^) employing a CRISPR/Cas9 system^34,35^ to select for positive recombinants (see “Materials and methods” section, Fig. S1).

The result was an engineered *E. coli* ΩacrB::PAmCherry1 strain (TV001) expressing a labelled version of AcrB under its native regulatory system (Fig. 1a)^36,37^. The AcrB-PAmCherry1 fusion retains its native function as well, which was verified by an antibiotic susceptibility test (see “Materials and methods” section, Fig. S2).

PAmCherry1 is a photo-activable chromophore which emits fluorescence when excited with green/yellow light, upon conversion with ultraviolet light, until it permanently bleaches. This feature makes it suitable for molecular counting. As such, by applying PALM to our bacterial strain we were able to count endogenous AcrB instances in both planktonic and biofilm associated bacteria.

**Figure 2:**
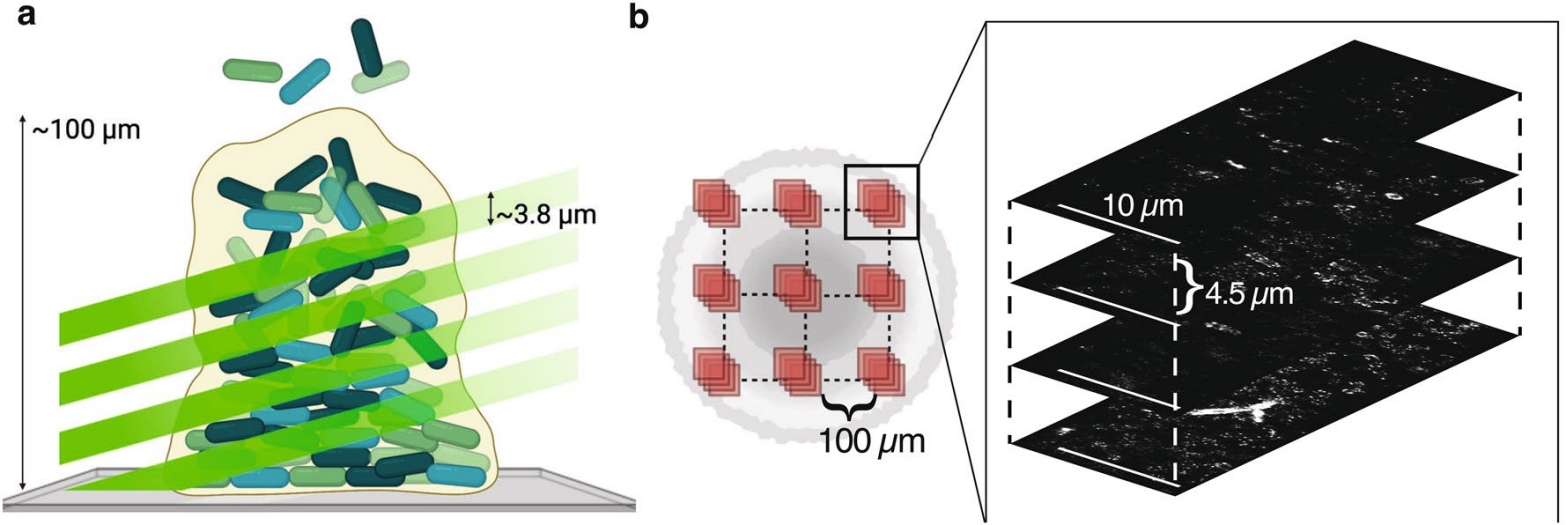
(**a**) Schematic of the single-objective light-sheet used to optically scan the biofilm volume to increase the signal-to-background level and enable single-molecule localization deep inside the biofilm. (**b**) Schematic of FOV positioning when imaging biofilms. A total of 36 FOVs (10 × 40 µm^2^, inset) were acquired for each sample, in a 3 × 3 × 4 grid. Imaged regions were separated by ~ 100 µm on the XY-plane. Each region on the XY-plane was imaged at four different depths, separated by 4.5 µm vertically, obtaining a 3D map of the AcrB distribution in the region. Created with BioRender.com.

Measuring AcrAB-TolC levels of expression in bacterial biofilms requires single molecule sensitivity in a thick sample. In fact, a biofilm is a three-dimensional mesh, growing on a surface through different maturation stages up to 100–200 μm thickness (Fig. 2a), where bacteria display different gene expression patterns in space and time. Its thickness poses challenges for performing PALM due to the poor signal-to-background with increasing in depth. In recent years, new “single objective” light sheet microscopy techniques have been developed to achieve high resolution in thick samples gaining direct access to the sample, that was limited in traditional dual-objective light sheet configurations. Single objective configurations are based on oblique plane microscopy (OPM), where a primary objective is used to illuminate the sample and collect the emitted signal, while secondary and tertiary objectives are used to remotely focus the oblique plane on the detector^38^. These configurations demonstrated high flexibility and applicability to samples over a wide range of spatial dimensions. In particular, high spatio-temporal resolution was demonstrated through DaXi, achieving 450 nm lateral and 2 mm axial resolution in a large volume of 3000 × 800 × 300 μm with low magnification objective (20×)^39^. The same group recently demonstrated full resolution maintained in OPM also with high NA, high magnification objectives (up to 100× immersion), thus opening the way to fast, high-resolution light sheet microscopy^40^. Also, several implementations of OPM have demonstrated SMLM on different spatial scales, from cells to small organism models^38^. All these techniques however rely on complex customized remote focusing configurations, comprising customized remote objectives used to reconstruct the image of the oblique plane on the detector. A much simpler approach could be to exploit highly inclined illumination out of a TIRF objective, demonstrated by Tokunaga et al. and named HILO^41^, to produce a produce a sheet of light propagating at high angle inside the sample. By displacing the objective along the optical axis, it is possible to optically scan the sample, by using the same objective to acquire the emitted signal without the need to tilt the image plane. Moreover, we recently proposed a simple customization of the excitation beam shape to reduce the thickness of the refracted beam to few microns, with a significant improvement of the signal-to-background and the number of localizations in PALM/STORM imaging^29^.

Our optical system allows for precise setting of both the angle of propagation of the excitation light sheet and its thickness according to the experimental needs, thanks to an adjustable aperture placed in a plane conjugated with the image plane of the objective and a motorized system for the controlled displacement of the excitation beam from the objective optical axis to impose the optimal angle and divergence to the inclined light sheet^29^ (Fig. S3). For its application to bacterial biofilm, we found that the optimal tradeoff between signal-to-background and dimension of the field of view (FOV = 10 × 40 μm^2^) is achieved with the light sheet inclined by an angle of 69°, with a consequent thickness of 3.8 μm (see “Imaging setup” in “Materials and methods” sections).

First, super-resolved images of single PAmCherry1in planktonic TV001 strain bacteria seeded on glass coverslip (“Materials and methods” section, Fig. S4) were obtained, with about 20 nm localization accuracy. By counting the number of localizations per cell we could directly quantify the level of expression of the pump within bacterial cells (quantification method will be detailed below in the manuscript). We tested our method by inducing AcrAB-TolC overexpression through exposure to 0.8 mg/ml sodium salicylate^9^. Figure S5 shows histograms of the number of localizations per cell for non-treated (control) and treated bacteria respectively. The significant difference between quantifications in the two conditions validates the method, thus making it accountable to directly monitor AcrAB-TolC levels of expression (see “Materials and methods” section).

Then, by scanning the light sheet along the optical axis of the objective we achieved single-molecule sensitivity deep inside the biofilm (Fig. 2a).

Before imaging bacterial biofilms, we verified the capability of our optical system to localize single chromophores within thick samples. First, we characterized the optical system by imaging 100 nm fluorescent beads embedded in 2% agarose gel (“Materials and methods” section for sample preparation, and Supplementary File_1). There, we reported a localization accuracy ranging from 3.1 to 8.3 nm with increasing depth from the glass surfaces up to 100 μm (Fig. S6). Then, to test the localization accuracy of our system with PAmCherry emission properties, we created a “synthetic biofilm” model sample, where planktonic *E. coli* TV001 bacteria were uniformly incapsulated in an agarose film made to match the optical density (OD) of real biofilms. On this sample we could localize fluorescently labelled AcrB up to 100 μm, with a localization accuracy ranging from 23 to 31 nm with increasing distance from the glass coverslip (as shown in Fig. S7).

Through this optical system, we investigated the expression of endogenous fluorescently labelled AcrB in *E. coli* TV001 at different time points over the course of biofilm growth from planktonic bacteria on glass coverslip, to 24 and 48 h grown biofilms (see “Materials and methods” section, Fig. S8).

Bacterial biofilms were imaged at different planes, separated by 4.5 um, to map AcrB expression level axially, and at different lateral positions within the same sample, by following the scheme depicted in Fig. 2b.

Stacks of images were acquired (Supplementary File_2) as described in “Materials and methods” section and processed with the ImageJ^42,43^ plugin ThunderSTORM^44^ to extract AcrB localizations. As we already mentioned, PAmCherry1 is well suited for molecular counting due to its photochemical behavior, where the switch from a non-fluorescent to a fluorescent state is irreversible and followed by permanent bleaching. Moreover, PAmCherry1 and its derivatives have been characterized as displaying negligible levels of stochastic blinking even compared to other irreversibly photoactivatable fluorescent proteins^45–47^. However, an individual PAmCherry1 can emit fluorescence for longer than the frame integration time, which would result in it being detected in multiple consecutive frames, leading to overcounting. To compensate for this, localization maps were processed through the “merge” function in ThunderSTORM so that molecules appearing within a radius of 30 nm from each other in consecutive frames were counted as one. Conversely, the risk that multiple different copies of PAmCherry1 located at a shorter distance from each other could be activated and imaged at the same time, and subsequently merged, was considered negligible as the intensity of the 405 nm laser was finely adjusted to promote the activation of only 0–2 fluorophores per cell at any given moment.

Further quantifications were performed through a dedicated custom MATLAB pipeline. First, each reconstructed image was segmented to define the total area populated by bacteria with respect to the biofilm matrix (Fig. S9a, “Materials and methods” section). After segmentation, we corrected for a “base level” fluorescence signal which did not depend on PAmCherry activation, that was quantified through stacks of frames acquired without activation through 405 nm prior to each measurement (see “Materials and methods” section, Fig. S9b for details). Finally, the density of fluorophores was calculated over the segmented area as the number of PAmCherry localizations per μm^2^.

**Figure 3:**
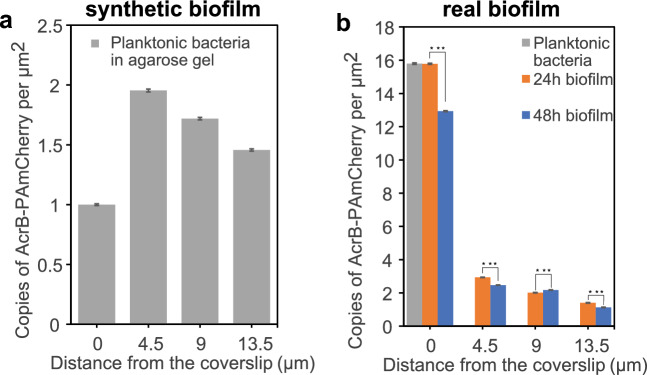
(**a**) Axial density profile of AcrB-PAmCherry in the synthetic biofilm. Protein density is measured at various distances from the glass-sample interface (0, 4.5, 9 and 13.5 µm), normalized on the value at 0 µm. Each value is the weighted average over 9 different FOV, laying at the same depth, for three independent biological replicates. Error bars are error of the weighted average. (**b**) Axial density profile of AcrB-PAmCherry in real biofilms. Data was corrected by taking into account values in (**a**) as reported in the main text and in Supplementary Materials. Protein density is measured at various distances from the glass-sample interface (0, 4.5, 9 and 13.5 µm), normalized on the value at 0 µm. Each value is the weighted average over 9 different FOV, laying at the same depth, for three independent biological replicates. Error bars are error of the weighted average. Gray bar is protein density in planktonic bacteria, orange bars are protein densities in 24 h grown biofilms and blue bars are protein densities in 48 h grown biofilms. Significance are Student’s t-test p-values calculated with the OriginLab software.

Further, the “synthetic biofilm” model sample described before was imaged and analyzed by following the same imaging scheme adopted for bacterial biofilms (Fig. 2b) to quantify possible impact on the density quantification rising from the illumination geometry. The measured density profile of the synthetic biofilm is reported in Fig. 3a. As bacteria are distributed uniformly inside the agarose gel, the differences in protein density could be due to either geometrical features of the sample architecture or to the optical response of the system. First, at the interface between the biofilm and the glass coverslip (0 μm) bacteria are mostly lying horizontally (analog to what we observe in “real” biofilms), with the major axis aligned with the coverslip surface, while at increasing distances from the base layer bacteria tend to be oriented isotropically. This leads to an increased bacterial area observed at the glass surface and a consequent decrease in the protein density compared to what is measured in deeper layers of the biofilm. On the other side, the negative trend of the protein density with depth inside the synthetic biofilm volume reflects the negative trend in the signal-to-background with depth (Fig. S10) which leads to a gradual decrease of the number of localized proteins. Quantification of protein density at different depths in the “synthetic biofilm” was then used to correct the protein densities measured in biofilms, as described in detail in the “Materials and methods” section and Fig. S11.

Figure 3b shows AcrB corrected densities at different depths for 24 h (orange) and 48 h (blue) grown biofilms respectively. The ground level (0 μm) is also compared with AcrB densities in planktonic bacteria (grey). While we do not observe a significant difference in the pump densities between different FOVs laying at the same depths, we observed that the average density of AcrB protein (calculated over 9 FOVs at same depth) is highest at the base layer of biofilms in contact with the glass coverslip (0 μm), where it is comparable to that observed in planktonic bacteria, while it drops sharply with increasing distance from the biofilm-coverslip interface, with the protein level vanishing over ~ 13.5 μm from the glass surface. Moreover, by quantifying the AcrB densities at different heights inside the biofilm over time, for 24 and 48 h grown biofilm, we observed an overall significant decrease of AcrB expression during biofilm growth.

## Discussion

In this work, we developed an innovative optical approach to achieve single-molecule sensitivity within three-dimensional thick samples and we applied it to the study of bacterial biofilms. By combining volumetric PALM with precision genome editing we could achieve, to the best of our knowledge, the first quantification of endogenous AcrB EPs expression level in *E. coli* biofilms, showing that its distribution varies largely in depth within the volume of biofilms and during biofilm maturation. The expression level of AcrB is highest at the base layer of biofilms, where it is comparable to that observed in planktonic bacteria, while it drops sharply with increasing distance from the biofilm-coverslip interface, eventually vanishing above about 13 μm of depth inside the biofilm volume. We also observed an overall decrease of AcrB expression over time during biofilm growth. Both these findings are in accordance with what observed by De Kievit et al. regarding biofilm distribution and time dynamics of the efflux pump complex MexAB-OprM^48^, a *Pseudomonas aeruginosa* homolog of the AcrAB-TolC complex. The spatial distribution that we found might relate to the different capacity of bacteria within the biofilm community to counteract external threats, with the “front line” exposed to the glass surface being more reactive, with much higher levels of EPs expression. Indeed, the decreasing EPs expression level with depth would implicate the presence of a “biofilm core” composed by bacteria with low metabolic activity as hypothesized previously^49^.

The development of our methodology opens the way to studies on biofilm growth and homeostasis and, importantly, to the investigation of how biofilms develop resistance to antibiotics. For example, our technique could be straightforward applied to the study of how bacterial biofilms respond to drug treatments and how environmental conditions can influence this process. Moreover, given the increasing interest in the application of super-resolution microscopy to thick samples, notably organoids and spheroids, we anticipate that our approach combining volumetric PALM imaging with genome editing can be fruitfully applied to a vast number of biological systems and will be of interest for a broad range of applications.

## Materials and methods

### Production of labelled *E. coli* strain via Cas9-assisted recombineering

We carried out the no-SCAR bacterial genome editing protocol by Reisch and Prather^32^ to replace the STOP codon on the original *acrB* gene on the chromosome of *E. coli* BW25113 with a codon-optimized, marker-free DNA sequence encoding for PAmCherry1 preceded by a short flexible peptide linker (Gly–Gly–Gly). Codon context^50–52^ was also taken into consideration in designing the recombination template to minimize unintended effects on the translation rate of the chimeric protein. We employed the web-based multi-objective optimization tool “Codon Optimization On-Line (COOL)” (http://cool.syncti.org/)^53^ to optimize both codon bias and codon context of our PAmCherry1 sequence for its expression in *E. coli*. The coding sequence in the recombination template was flanked by a 214-bp homology arm (HA) at the 5′ and a 272-bp HA at the 3′. The 5′ and 3′ HAs were homologous to, respectively, the last 214 bases of the native *acrB* gene on the chromosome of BW25113 (excluding the STOP codon) and the first 272 bases downstream of the original STOP codon. The 5′ HA also contained a C-> T silent mutation at position 3129 of the *acrB* gene to disrupt the chosen PAM site for Cas9-mediated selection.

The recombination template, as shown in Fig. S1, was commissioned as a 1203 bp-long double-stranded DNA fragment (GeneStrand) to Eurofins Genomics (https://www.eurofinsgenomics.eu/), and then cloned into an adapted pFC20A plasmid via AQUA Cloning^54^ for amplification purposes.

Plasmid pCas9-CR4 (https://www.addgene.org/62655/) containing the *cas9* gene and plasmid pKDsgRNA-ack (https://www.addgene.org/62654/) containing the λ Red recombination genes *exo*, *beta* and *gam* as well as a single-guide RNA (sgRNA) expression cassette, were ordered from Addgene.

Plasmid pKDsgRNA-ack contains a sgRNA cassette designed for targeting the *ack* gene in *E. coli*. We retargeted this plasmid towards the PAM site in the *acrB* gene through site-specific mutagenesis (Quik Change kit, Agilent), obtaining plasmid pKDsgRNA-acrB.

pKDsgRNA-ack was PCR amplified with the following mutagenic primers:

FW: 5′-ATCAATGATGATCGACAGTAGTTTTAGAGCTAGAAATAGCAAG-3′

REV: 5′-TACTGTCGATCATCATTGATGTGCTCAGTATCTCTATCACTGA-3′

These two primers anneal to the same region of the plasmid, replacing the 20-bp long protospacer targeting *ack* with a new protospacer targeting *acrB*.

The PCR product was then digested with DpnI to remove the original pKDsgRNA-ack plasmid from the mix and directly transformed via heat shock in XL 10-Gold chemically competent cells (Agilent), which were then selected on LB-agar plates with added spectinomycin. As pKDsgRNA plasmids hold a temperature-sensitive origin of replication, all growth passages involving these plasmids were performed at 30 °C. Plasmid pKDsgRNA-acrB was then extracted from a few colonies via standard miniprep and the correct mutation was verified by sequencing.

After retargeting the sgRNA cassette, BW25113 bacteria were prepped for CRISPR/Cas9-assisted recombineering by transforming them sequentially with pCas9-CR4 and pKDsgRNA-acrB. It is crucial that these two transformations are performed in this exact sequence and at different times as both the *cas9* gene and the sgRNA cassette are under the control of a TetR-repressed operator. This transcription regulation system is extremely effective at maintaining a null expression level in the absence of the inductor (anhydrotetracycline, or aTC) but rely on the intracellular accumulation of the TetR repressor to block gene transcription. Since a single Cas9 molecule coupled with a single sgRNA is sufficient to induce a double-strand break on the DNA of a bacterium leading to cell death, it is crucial to prevent expression of both genes at the same time. The *tetR* gene is encoded on plasmid pCas9, therefore the transformation of this plasmid must happen before the transformation of pKDsgRNA to allow for the accumulation of the TetR protein inside cells.

First, BW25113 bacteria were made chemically competent with the calcium chloride method^55^; they were transformed with pCas9-CR4 via heat shock and then selected on LB-agar plates with added chloramphenicol. Cells harboring pCas9-CR4 were again made chemically competent with the same technique and transformed with pKDsgRNA-acrB. After a recovery step at 30 °C, half of the transformation mix was plated on LB-agar with added chloramphenicol and spectinomycin. The other half was plated on dishes with added aTC. As aTC is a transcription inductor of both Cas9 and sgRNA, a comparison between the number of colonies grown on these plates and in plates without added aTC was used to verify that the CRISPR/Cas9 selection system was working properly.

BW25113 bacteria harboring both pCas9-CR4 and pKDsgRNA-acrB were then grown in LB broth with the proper antibiotics until mid-log phase, at which point arabinose was added at a final concentration of 1.2% in order to induce the λ Red genes in plasmid pKDsgRNA-acrB. After 15 min of induction, the cells were made electrically competent using glycerol/mannitol density step centrifugation as described by Warren^56^.

Electrically competent cells were immediately transformed with 1500 ng of (PCR-amplified and purified) linear GeneStrand via electroporation, recovered at 30 °C, and then plated on LB-agar containing chloramphenicol, spectinomycin and aTC. Colonies were screened for positive recombination via colony PCR with primers gStrand_FW and gStrand_REV, and then verified by sequencing.

The pCas9-CR4 plasmid (Addgene plasmid # 62655; http://n2t.net/addgene:62655; RRID:Addgene_62655) and the pKDsgRNA-ack plasmid (Addgene plasmid # 62654; http://n2t.net/addgene:62654; RRID:Addgene_62654) were gifts from Kristala Prather, Massachusetts Institute of Technology, Department of Chemical Engineering. All of the *E. coli* BW25113 strains used in this study were gifts from Laura Piddock, University of Birmingham, Institute of Microbiology and Infection.

### Antibiotic suceptibility test

We verified that AcrB-PAmCherry1 remained functional with a chloramphenicol susceptibility test. Chloramphenicol is a well-characterized substrate of AcrB^57^, and we found that the minimal inhibitory concentration (MIC) of chloramphenicol for TV001 was equal to the one for WT E. coli, and 4 times higher than the MIC for KO strains for either *acrB* or *tolC* (Fig. S2).

### Treatment with sodium salicylate

Planktonic bacteria TV001 were exposed to 0.8 mg/mL sodium salicylate for 2 h to induce AcrB overexpression. The shift between the two distribution is significant and accounts for the different level of expression of the pump in the two conditions.

### Imaging setup

We employed a custom-built microscopy setup with single-molecule imaging capabilities to perform quantitative PALM on *E. coli* TV001 cells in various conditions^29,58,59^. 405 nm and 532 nm diode lasers were employed to, respectively, photoactivate PAmCherry1 and excite the fluorescence of PAmCherry1 in its activated state. The same objective was used both to direct the excitation lasers on the samples and to collect fluorescence signals, and a multiband dichroic mirror (405/488/562/635 nm lasers BrightLine® quad-edge laser dichroic beamsplitter, Semrock) was employed to decouple the emitted signal from the excitation laser light and to direct the former towards an Electron Multiplying CCD camera with single-photon sensitivity. A detailed description of the optical setup is provided in Fig. S3a. A set of motorized translators (Fig. S3b) on our setup allows to finely set the inclination of the excitation beam by following the equation

$${\theta }_{r}= {\text{sin}}^{-1}\left(\frac{d}{{n}_{sample}{f}_{obj}}\right),$$

where *d* is the distance of the focused beam from the optical axis of the objective, *n*_*sample*_ is the refractive index of the sample and *F*_*obj*_ is the focal length of the objective; while the thickness of inclined beams can be regulated via an adjustable slit located on a plane conjugated with the image plane^29^ (BS in Fig. S3a).

### Preparation of planktonic bacteria and biofilm samples for imaging

#### Samples of planktonic bacteria were prepared in the following way

Bacteria (either WT *E. coli* BW25113 or TV001) were grown in M9 with added glucose (0.4%) overnight at 37 °C under vigorous shaking. The next day, 1 ml of culture was centrifuged (5000×*g* for 5 min), resuspended in a paraformaldehyde solution (4% PFA in PBS) and incubated overnight at 4 °C. The cells were then centrifuged (5000×*g* for 5 min) and resuspended in PBS twice to remove excess formaldehyde. 20 µl aliquots of fixed bacterial suspension were then fluxed in custom-built glass imaging chambers coated with poly-l-lysine and left to adhere to the coverslip for at least 20 min prior to imaging. A schematic illustration of these chambers is shown in Fig. S4^60^.

#### Biofilm samples were prepared in the following way

A starting bacterial culture was grown overnight in M9 medium with added glucose. The next day, 25 µl of the starting culture were inoculated in 1 ml of fresh M9 medium with added glucose previously placed on an open-ceiling imaging chamber (Fig. S8), assembled with a bottom coverslip coated with poly-l-lysine. The imaging chamber containing the inoculated medium was placed inside of a 100 mm sterile cell culture dish and incubated at 37 °C for either 24 or 48 h without any shaking. A custom humidifying system was assembled inside the incubator in order to minimize drying of the medium. After the incubation period, a biofilm layer was clearly visible at the bottom of the imaging chamber. The culture medium was very gently removed with a 1 ml syringe, paying attention not to detach the biofilm from the coverslip. Then, the biofilm was gently covered with 500 µl of a 4% PFA solution in PBS and incubated overnight at 4 °C. The fixing solution was delicately removed, and the biofilm was washed twice with 500 µl of PBS. Finally, the chamber was mounted on the microscope for imaging.

### Image acquisition

To accurately account for autofluorescence and, more generally, fluorescence signal that was unrelated with the emission of photoactivated PAmCherry1, two consecutive 1000-frames-long videos were acquired for each field of view (FOV). The first one was taken under illumination with 532 nm light but prior to photoactivation with 405 nm light; the second one was taken under simultaneous illumination with 532 nm (~ 700 W/cm^2^) and 405 nm light (0.7 W/cm^2^) in order to elicit photoactivation of PAmCherry1 over the course of the acquisition, 100 ms exposure time and 400 EMGain. The correction procedure through analysis of non-photoactivated frames is described in the next section.

For each biofilm sample, PALM videos were acquired in a total of 36 FOVs, as exemplified in Fig. 2b,c, in a 3 × 3 × 4 three-dimensional grid where FOVs were separated by 100 µm horizontally and by 4.5 µm vertically, which was allowed by the reduced excitation light-sheet thickness of ~ 3.8 µm. The order in which FOVs were imaged was designed in such a way as to avoid potentially exposing yet-to-be-imaged FOVs to any laser light coming from previous acquisitions.

The samples of planktonic bacteria consisted in a single layer of cells stuck on the surface of a glass coverslip, as such, only a single plane was imaged with 9 FOVs for each sample. The quantification was carried out on data from 3 independent replicates.

### Image reconstruction, segmentation and correction of localization quantification

PALM videos were processed with ThunderSTORM^44^, an open-source plugin for ImageJ^42,43^ for the analysis and visualization of data acquired through PALM/STORM, to reconstruct super-resolved localization maps. Localization maps were filtered with an empirically determined filter (offset > 0.5 & offset < 6 & sigma < 300 & uncertainty < 60 & bkgstd < 2.3 & intensity < 400) to remove outliers. Then, localization maps were processed through the “merge” function in ThunderSTORM so that molecules appearing within a radius of 30 nm from each other in a timeframe of 10 consecutive frames were counted as one, to compensate for emission of PamCherry for longer than the exposure time that would lead to overcounting.

As both planktonic bacterial samples, especially biofilms, are not uniformly distributed in space within the FOV but tend to form clusters of cells alongside empty areas, a custom MATLAB pipeline was used to define areas populated by cells and to segment the images around those. We also observed a small subpopulation of bacteria emitting an especially high amount of fluorescence signal that was completely independent from photoactivation (which we named “torches”), which were excluded from our analysis (Fig. S9a). The segmentation algorithm works in the following way: a circular area 1 μm in diameter is selected around each localized emitter in an image, and each pixel of the selected area is assigned a value of 1. When the circular areas around multiple emitters overlap, their values are summed together in the pixels making up the intersection. At the end, each pixel of the image is associated with a value that indicates the local density of fluorophores, and the image can be segmented applying lower and upper thresholds on these values. The thresholds in this study were determined empirically to provide the most accurate segmentation upon visual examination and correspond to 0 and 150 (Fig. S9a). All codes are available at https://github.com/TVignolini/.

After segmentation, the first of each PALM video pair acquired for each FOV was analyzed to estimate a “base level” of spurious signals that was then used to correct the subsequent measurement. The number of localizations per frame of the first video (located within the segmented area obtained from the subsequent PALM video) was plotted over the number of frames and fitted with an exponential function (Fig. S9b). The fitted function was then integrated for x between frame 1001 and 2000. The resulting value, representing the predicted number of localizations in the second video that were not dependent on photoactivation, was subtracted from the total amount of localized molecules in the second video.

All the MATLAB codes used for the quantification correction are available at https://github.com/TVignolini/.

### Preparation and imaging of agarose gel with embedded fluorescent beads

2% agarose was prepared from agarose powder (SIGMA A9539) and Milli-q ultrapure water. The solution was melted in a microwave oven and kept at about 60℃ by immersion in a thermal bath. Fluorescent TetraSpeck beads, 100 nm diameter (T7279, ThermoFisher), were diluted 100 times in warm 2% agarose, by careful mixing. A final volume of 100 µl of this solution was sandwiched between a glass microscope slide and a 24 × 32 mm glass coverslip to obtain a final agarose film with a thickness of approximately 130 μm. The bead concentration was adjusted to distinguish and localize single beads through PSF fitting. Images of fluorescent beads were acquired using the imaging setup described above, with 50 ms exposure time and 100 EMgain, upon excitation at 552 nm.

### Synthetic biofilm preparation and imaging

To correct for biases in protein quantification due to imaging at varying depths, we prepared a calibration sample in the following way.

*E. coli* TV001 were grown overnight in M9 medium with added glucose. The next day, the absorbance (OD_600_) was measured, cells were centrifuged and resuspended in PBS to a final OD_600_ of 5. The bacterial cell suspension was mixed 1:1 with a low melting point agarose 2.4% solution at 40 °C. After thoroughly pipetting the mixture, 100 µl were dropped in the center of a warm glass slide, gently covered with a warm 24 × 32 mm glass coverslip, and let solidify at room temperature for 20 min prior to imaging.

Imaging and image analysis of these samples was performed exactly as described for biofilms. The calculated localization densities for each imaged plane (0, 4.5, 9 or 13.5 µm from the coverslip/sample interface) were then normalized on the value measured at the coverslip/sample interface (Fig. S11b). Three biological replicates of the calibration sample were prepared and imaged.

Localization densities measured in biofilms (Fig. S11a) were then corrected (Fig. S11c) by dividing each one for the normalized value of the corresponding plane measured in the calibration samples.

### Supplementary Information

Supplementary Information 1.

Supplementary Information 2.

Supplementary Information 3.

## Data Availability

The datasets generated and/or analysed during the current study are available in the GenBank repository, Accession Numbers PP850105, PP850106 and PP850107.
