# Supplementary figures and images for: Highly inclined light sheet allows volumetric super-resolution imaging of efflux pumps distribution in bacterial biofilms
Source: Sci Rep. 2024 Jun 5;14:12902. doi: 10.1038/s41598-024-63729-x (PMC11153600; doi:10.1038/s41598-024-63729-x)

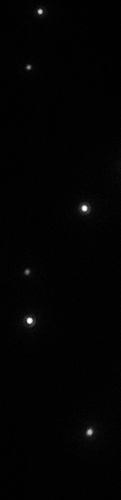

Supplement: Supplementary file 1 — Supplementary Information 1. [file 41598_2024_63729_MOESM1_ESM.zip › Supplementary file_1.tif]
