# Supplementary material for: Highly inclined light sheet allows volumetric super-resolution imaging of efflux pumps distribution in bacterial biofilms
Source: Sci Rep. 2024 Jun 5;14:12902. doi: 10.1038/s41598-024-63729-x (PMC11153600; doi:10.1038/s41598-024-63729-x)
Supplement: Supplementary file 3 — Supplementary Information 3. [file 41598_2024_63729_MOESM3_ESM.pdf]

Supplementary Information

Highly Inclined Light Sheet allows volumetric super-resolution imaging of efflux pumps distribution in bacterial biofilms

Vignolini T.[1,2,#,*], Capitanio M.[1,2], Caldini C.[1,2], Gardini L.[1,3,*,4] and Pavone F.S.[1,2,4]

[1] European Laboratory for Non- Linear Spectroscopy, LENS, Via N. Carrara 1, 50019 Sesto Fiorentino, Italy

[2] Department of Physics and Astronomy, University of Florence, Via G. Sansone 1, 50019 Sesto Fiorentino, Italy

[3] National Institute of Optics, National Research Council, Via N. Carrara 1, 50019 Sesto Fiorentino, Italy

#current affiliation: Institut Pasteur, Université Paris Cité, Parasite RNA Biology Group, Paris, 75015, France

[*] corresponding authors:
Gardini L., gardini@lens.unifi.it
Vignolini T., tiziano.vignolini@pasteur.fr

[4] these authors contributed equally.

Supplementary Figures

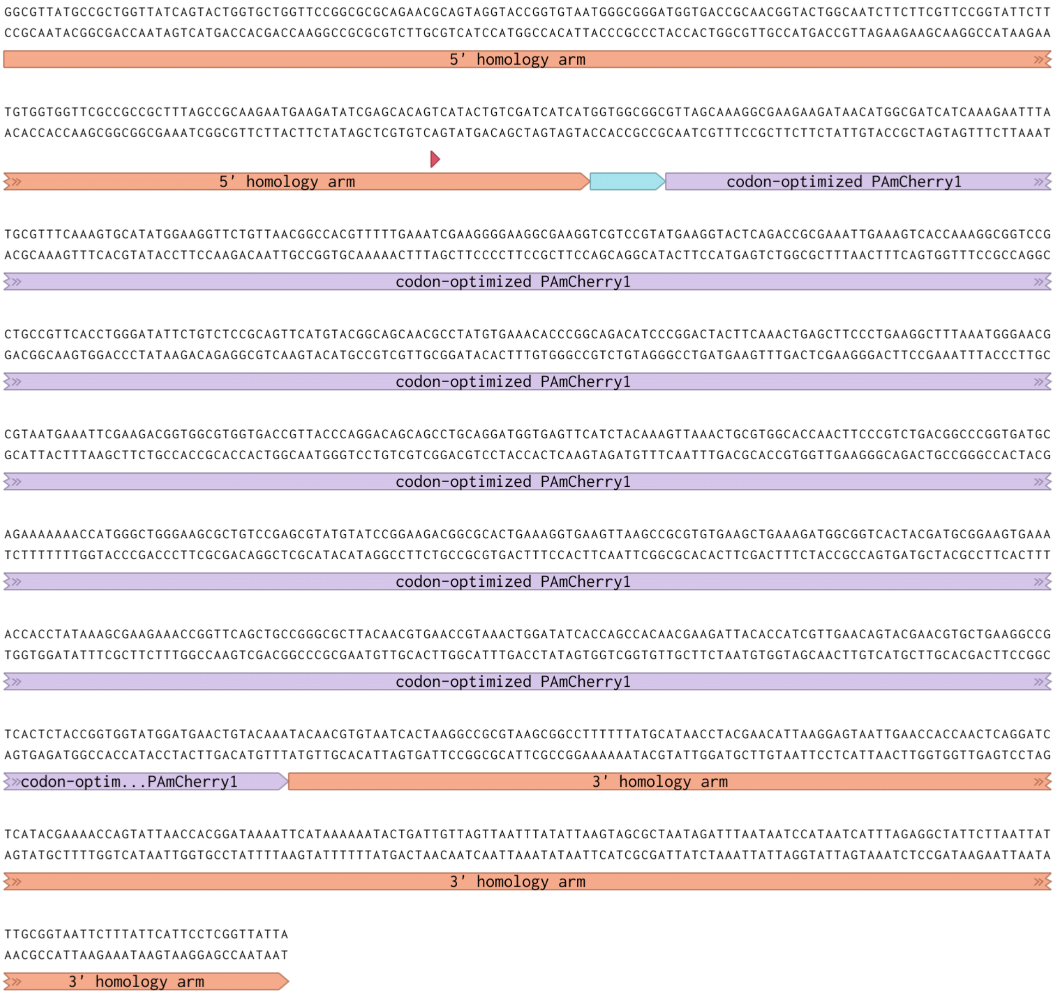

Figure S1. Sequence of the GeneStrand used as a recombination template. The red arrowhead indicates the silent C->T mutation in position 2139. The cyan segment indicates the sequence encoding for the Gly-Gly-Gly linker peptide.

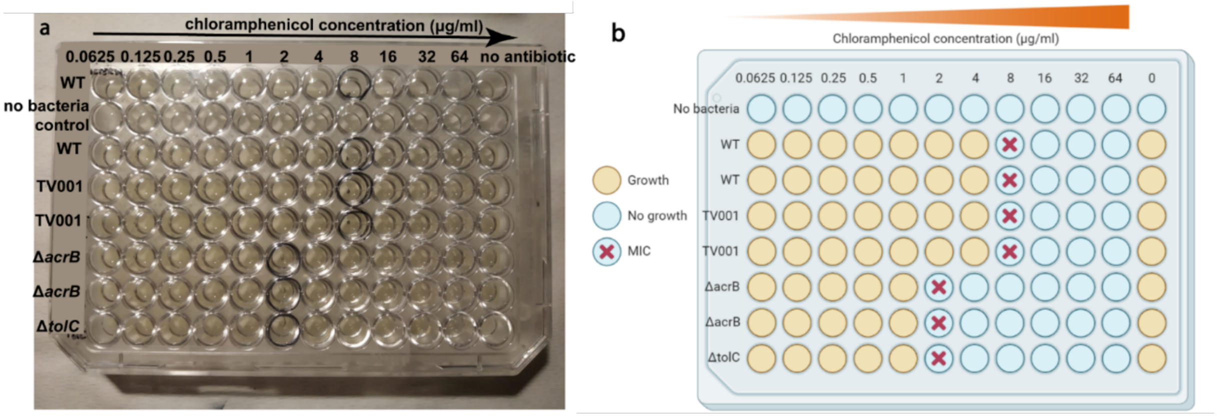

Figure S2. Antibiotic susceptibility test. **a)** Photo of the 96-well plate used for the microdilution experiment. Each well contains LB medium with a chloramphenicol concentration indicated by the respective column header, and a bacterial inoculum indicated by the row. Black marker circles highlight the minimal

concentration of chloramphenicol for each row at which no bacterial growth is visible. The MIC for both TV001 and wild-type bacteria is 8 µg/ml, while the MIC for both efflux pump mutants is 2 µg/ml. The image was taken after an overnight incubation (~16 hours). **b)** Schematization of the results.

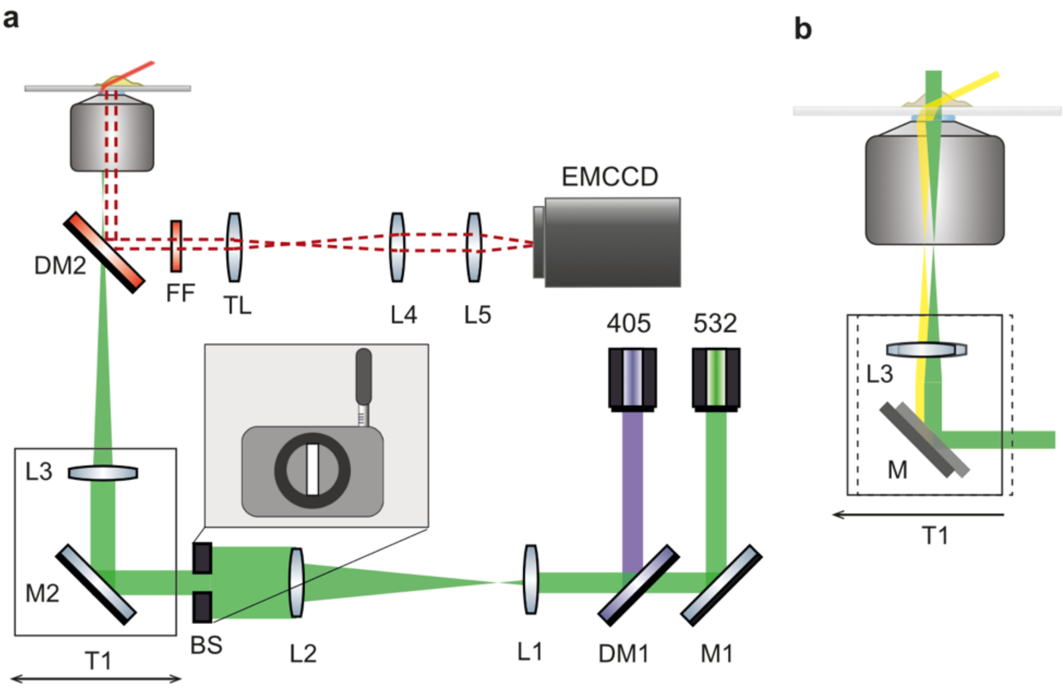

Figure S3. Schematic of the custom imaging setup. **a)** 532 nm and 405 nm diode lasers are directed along the same optical axis by specific dichroic mirror (DM1) and are magnified through a 10x telescope (L1, L2) before being focused on the back focal plane of a TIRF Nikon 60x oil immersion objective with a numerical aperture of 1.49. An adjustable slit (BS, inset) is placed on a conjugated plane with the sample to set the size of the excitation beam [1]. The objective is placed in inverted configuration, and the fluorescence signal is separated from the excitation through the dichroic mirror DM2, filtered through a bandpass filter (FF) and directed towards the detection components. The 60x fluorescence image generated after the tube lens (TL) is magnified 3 more times through telescope lenses L4, L5 and projected on an Andor iXon X3 EMCCD camera. The objective is mounted on a piezoelectric translator to adjust the focus and the sample is mounted on a piezoelectric stage with two independent axes of movement. **b)** The inclination of the excitation beam can be controlled by moving the motorized translator T1, which shifts both the M2 mirror and the L3 lens sideways with respect to the optical axis of the objective.

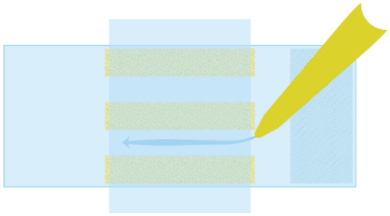

Figure S4. Imaging chamber for planktonic bacteria. The imaging chamber is composed of a rectangular glass coverslip attached on a microscope slide through thin strips of double sticky tape, which also serve as delimiters between separate channels. Each channel has a volume of approximately 20 µl. Samples and solutions are fluxed into the channels by pipetting through one of the open ends.

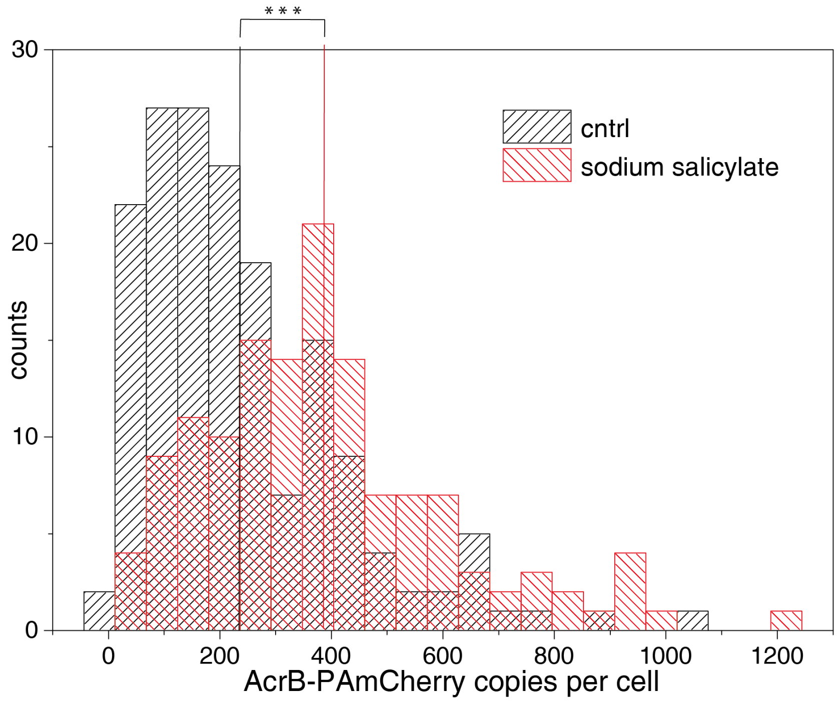

Figure S5. Results of sodium salicylate treatment. Distributions of pumps counting per cell for control bacteria (non-treated bacteria in LB) and bacteria treated with 0.8 mg/mL sodium salicylate for 2h. Continuous black and red lines are averages and standard errors: 238 ± 14 (control), 386 ± 19 (treated). $P < 0.001$ according to Student's t-test.

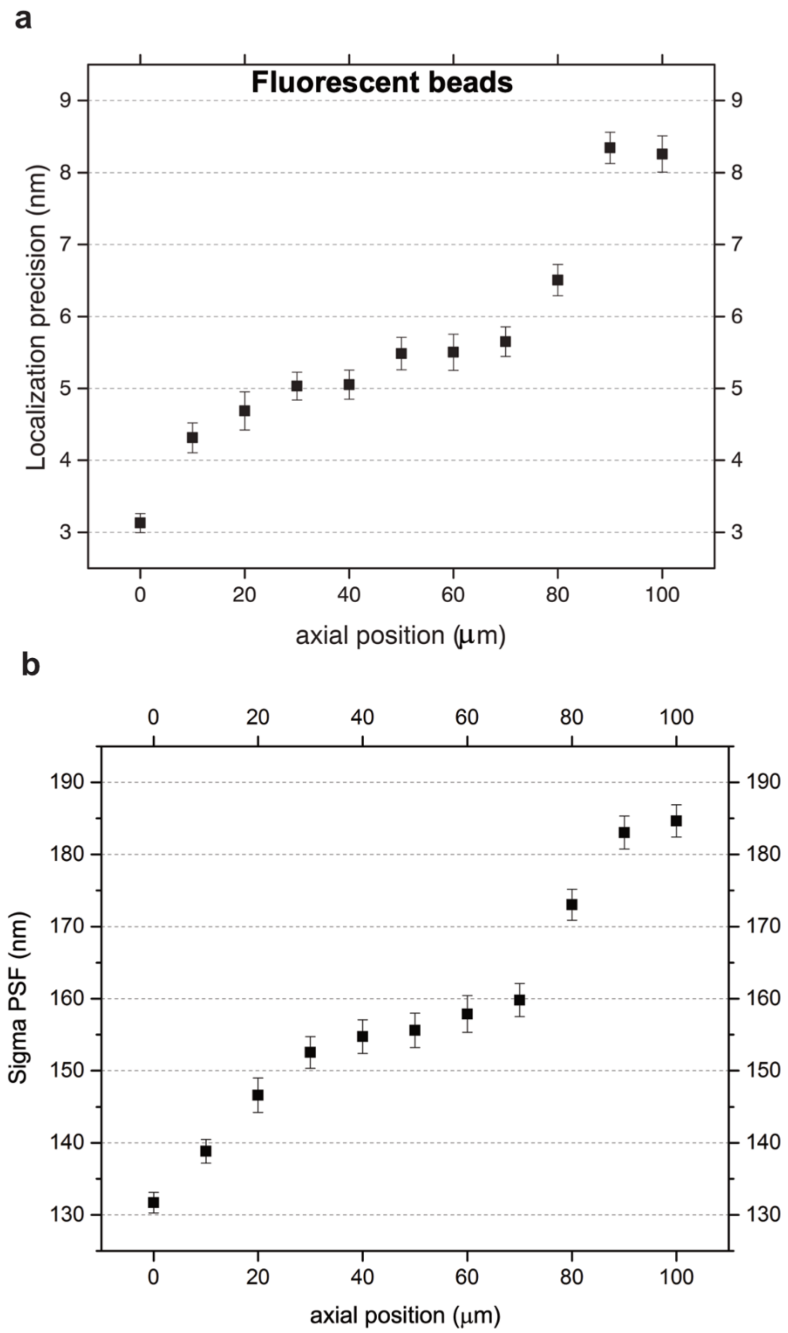

Figure S6 Localization precision and sigma PSF for 100 nm fluorescent beads as a function of the axial position. 0 µm corresponds to the glass coverslip surface. Images of beads at each depth were acquired as detailed in Materials and Methods and the beads position was localized with opensource ImageJ software ThunderSTORM [2]. ThunderSTORM performs fitting of the intensity profile of the single beads with a Gaussian function to extract the coordinates of the central peak. Another parameter given by fitting is the "Localization uncertainty", which is calculated from the Thompson equation[3]

$$\sigma_{\mu} = \sqrt{\frac{s_i^2}{N} + \frac{a^2/12}{N} + \frac{8\pi s_i^4 b^2}{a^2 N^2}}$$

where $\frac{s_i^2}{N}$ is the photon noise (being s_i the width of the intensity distribution), $\frac{a^2/12}{N}$ is the effect of the finite size of the pixel size of the image a, and $\frac{8\pi s_i^4 b^2}{a^2 N^2}$ is the effect of the background (being b the standard deviation of the background). Panel **a)** shows average values of Localization uncertainty as a function of depth. Dots are

average ± s.e., 15<N<35 for each axial position. **b)** Shows averages of sigma of the PSF as a function of depth. Dots are average ± s.e., 15<N<35 for each axial position.

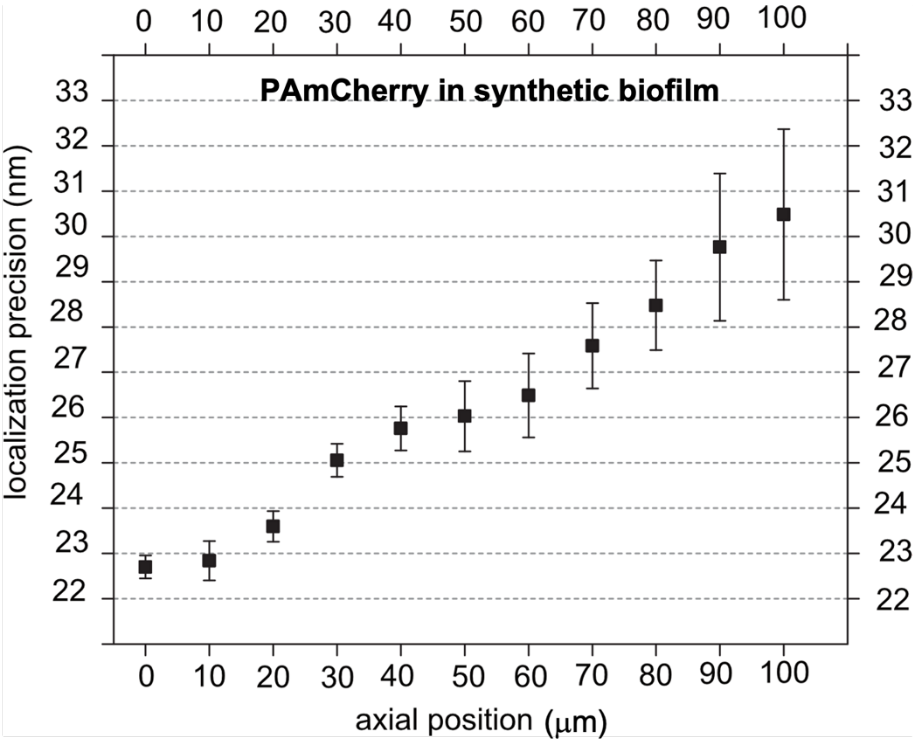

Figure S7 Localization precision as a function of depth for PAmCherry in the "synthetic biofilm" sample. Localization precision are "Uncertainty" values given by the reconstruction software "ThuderSTORM" as described in previous FigureS7, calculated by Thompson equation [3]. Dots are weighted average ± weighted error. 700<N<50, for each axial position.

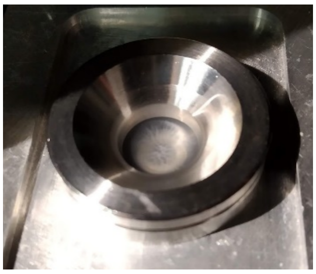

Figure S8. Biofilm imaging chamber. Biofilm sample mounted on an imaging chamber.

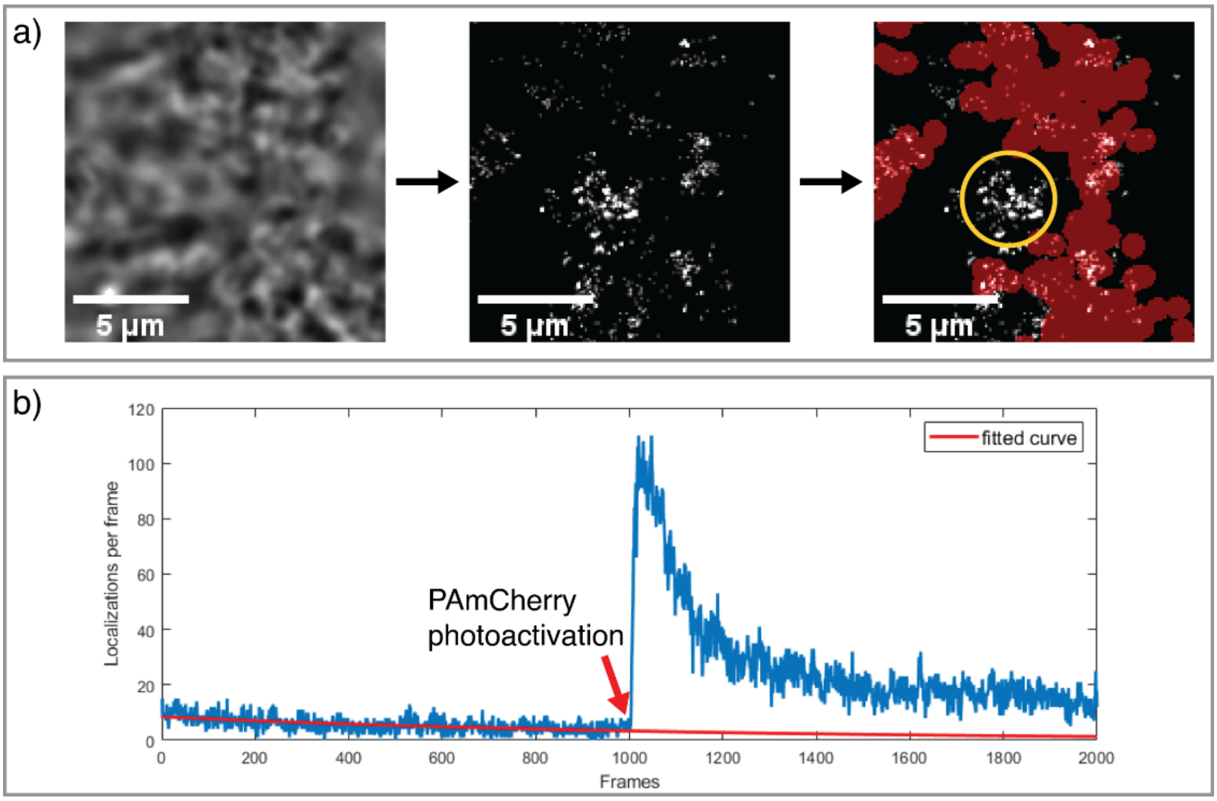

Figure S9. Schematic of the imaging and analysis procedure. a) A bright field image is taken as a reference prior to acquiring a PALM video. The video is processed with ThunderSTORM, obtaining a super-resolved fluorophore map. The map is segmented by an automated algorithm that identifies areas populated by bacteria (in red) and excludes "torches" (yellow circle) based on a localization density threshold. **b)** Graph showing the number of localized molecules per frame in the segmented area of a typical PALM video. The first 1000 frames are acquired prior to photoactivation of PAmCherry1, after which the 405 nm illumination is turned on. An exponential function (red curve) is fitted on the number of localizations per frame over the first 1000 frames. It is then integrated over frame 1001 to 2000, and the resulting value is subtracted from the total amount of localized molecules, as it indicates a base number of localizations that are independent from photoactivation and therefore are not due to PAmCherry1 fluorescence.

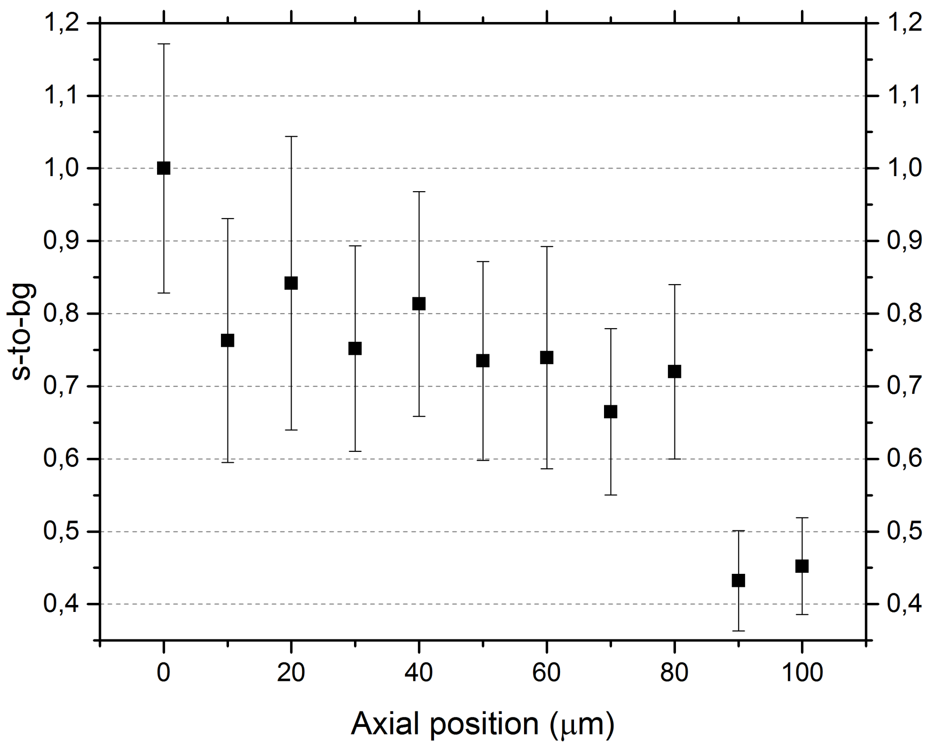

Figure S10 Signal-to-background ratio of 100 nm fluorescent beads in 2% agarose gel. Signal-to-background is calculated as $I_{max} - I_o/I_o$, where I_o is the offset level of the Gaussian fit performed by ThinderSTORM, that represents the baselevel of the intensity profile given by the background level in the proximity of the emitter, and I_{max} is the intensity of the peak. Intensity values are normalized on axial position= 0 μm value. As expected, the s-to-bg is decreasing with depth.

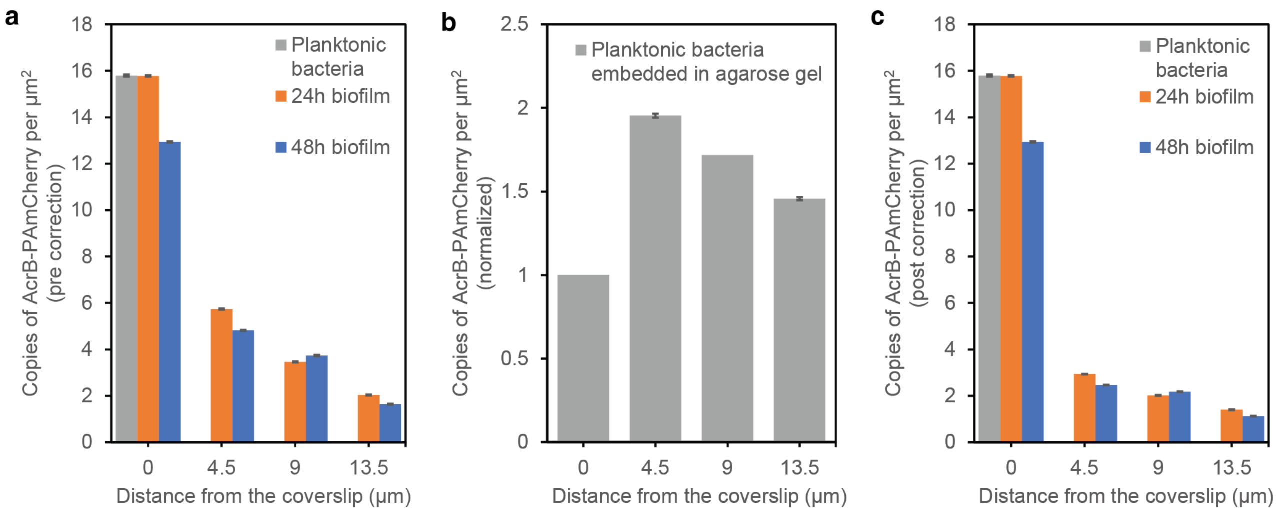

Figure S11. Density distributions as a function of depth and maturation stage. AcrB-PAmCherry1 density in cells measured in biofilms at various distances from the coverslip before correction **a)**, measured in calibration samples **b)**, and measured in biofilms and then corrected based on the results from calibration samples **c)**.

Supplementary References

[1] L. Gardini, T. Vignolini, V. Curcio, F. Saverio Pavone, and M. Capitanio, “Optimization of highly inclined illumination for diffraction-limited and super-resolution microscopy,” *Opt. Express*, vol. 31, no. 16, p. 26208, 2023, doi: 10.1364/OE.492152.

[2] M. Ovesný, P. Křížek, J. Borkovec, Z. Švindrych, and G. M. Hagen, “ThunderSTORM: a comprehensive ImageJ plug-in for PALM and STORM data analysis and super-resolution imaging,” *Bioinformatics*, vol. 30, no. 16, pp. 2389–2390, Aug. 2014, doi: 10.1093/BIOINFORMATICS/BTU202.

[3] R. Thompson, D. Larson, W. W.-B. journal, and undefined 2002, “Precise nanometer localization analysis for individual fluorescent probes,” *Elsevier*, Accessed: Feb. 10, 2020. [Online]. Available: https://www.sciencedirect.com/science/article/pii/S000634950275618X
